# The Role of  the Distal Splenorenal Shunt in the Sclerotherapy Era?

**DOI:** 10.1155/1994/37406

**Published:** 1994

**Authors:** J. Michael Henderson

**Affiliations:** Department of General Surgery The Cleveland Clinic Foundation 9500 Euclid Avenue Cleveland Ohio 44195 USA


Clearly it is essential to have an organized, experienced team in place. One needs an energetic endoscopist, an aggressive surgeon, a capable anesthesiologist and the rest of the supporting cast to undertake prompt diagnostic procedures, perform major surgery quickly, to provide nutritional support and to carry the patient through the post-operative period and a long term follow-up.

Despite all this praise, Orloff is not perfect. He makes the point that he is using the Child-Turcotte-Campbell system of grading the severity of cirrhosis[7], but he is not. In the Campbell modification it is necessary to determine whether the ascites is "easily controlled" or "difficult to control", decisions that require multiple observations. In Orloff's series ascites is graded on its preserved absence and volume immediately after a discussion. Furthermore, there are better methods of treating active bleeding than by infusing vasopressin.

One aspect of these data must be taken into serious consideration, i.e. "*The Wizard Phenomenon*". A wizard is defined as "one endowed with exceptional skill...and able to achieve something held to be impossible"[9]. Orloff's reported feats with EPCS surgery easily satisfy this definition. One must worry whether subsequent surgeons can equal Orloff's achievements, but this lis the way of the world. In all fields of human endeavor progress is made by incremental breakthroughs. On October 18, 1968, for example Bob Beamon made a prodigious longjump at the Olympics is Mexico City (8.90 m), almost 22 inches, longer than the existing world record, which had changed less than 8 inches in 33 years. In 1991, this new record, too, was broken[10]. Starzl *et al.*, performed the first liver transplant in a human in 1963 a unique occurrence thought then to be of little practical value, and now, thirty years later, this procedure is performed as standard therapy at hundreds of hospitals. EPCS has been performed by many surgeons for many years, perhaps, not so successfully as Orloff, but I predict that using a different time frame, surgeons will be able to perform this procedure as rapidly and effectively as the Wizard of San Diego.

After considering all these data I think that EPCS may be the one true treatment of actively bleeding varices and that Orloff is its prophet.

### *References*

1. Orloff, M. J., Orloff, M. S., Rambotti, M. and Girard, B. (1992) Is portal-systemic shunt worthwhile in Child's Class C cirrhosis? *Ann. Surg.*, **216**, 256–268.
2. Conn, H. O. (1994) Subclinical hepatic encephalopathy. In Hepatic Encephalopathy: Syndromes and Therapies, Ed. Conn, H. O. and Bircher, J. Medi-Ed Press, East Lansing, Michigan, p. 27–42.
3. Zieve, L., Doizaki, W. M. and Zieve, F. J. (1974) Synergism between mercaptans and ammonia or fatty acids in the production of coma: a possible role for mercaptans in the pathogenesis of hepatic coma. *J. Lab. Clin. Med.*, **83**, 16–28.
4. Conn, H. O. and Lieberthal, M. M. (1979) The Hepatic Coma Syndromes and Lactulose, Williams & Wilkins, Baltimore, Maryland, p. 28–29.
5. Mutchnick, M. C., Lerner, E. and Conn, H. O. (1974) Portal-systemic encephalopathy and portacaval anastomosis: a prospective controlled investigation. *Gastroenterology*, **66**, 1005–1019
6. LeVeen, H. H. (1985) The LeVeen shunt. *Ann. Rev. Med.*, **36**, 453–469.
7. Campbell, D. P., Parker, D. E. and Anagnostopoulos, C. E. (1973) Survival prediction in portacaval shunt: a computerized statistical analysis. *Am. J. Surg.*, **126**, 748–751.
8. Orloff, M. J., Bell, R. H. Jr and Greenburg, A. G. (1986) Prospective randomized trial of emergency portacaval shunt and medical therapy in unselected cirrhotic patients with bleeding varices. *Gastroenterology*, **90**, 1754 (Abstract).
9. Webster Third New International Dictionary, 1966.
10. Progression of World Best Performances and IAAF Approved World Records, Eds. Wigley, J. and Pearce, J., Peckham, V. Co-ordinated by LOC of IAAF 3rh World Championships in Athletics, 1992.

Harold O Conn
Yale University School of Medicine
New Haven
Connecticut and D.V.A. Medical Center
West Haven, Connecticut 06516, U.S.A.

### THE ROLE OF THE DISTAL SPLENORENAL SHUNT IN THE SCLEROTHERAPY ERA?

### ABSTRACT

*Rikkers, L F., Jin, G., Burnett, D. A., Buchi, K. N. and Cormier, R. A. (1993) Shunt surgery versus endoscopic sclerotherapy for variceal hemorrhage: Late results of a radomized trial. The American Journal of Surgery, 165, 27–33.*

**Between September 1982 and April 1988, 60 Cirrhotic patients with prior variceal hemorrhage were randomized to undergo the placement of an elective shunt (distal splenorenal: 26; nonselective: 4) or long-term endoscopic sclerotherapy ($n=30$). Eighty-six percent of patients had alcoholic cirrhosis, and 33% were classified as Child's class C. After a mean follow-up of 87 months, 60% of patients undergoing sclerotherapy and 17% of shunt patients experienced rebleeding ($p < 0.001$). Shunt patients have survived longer than those who had sclerotherapy (6-year survival rates of 53% and 26%, respectively; $p < 0.05$). In part because of the wide geographic distribution of patients, only 4 of 13 patients in whom sclerotherapy failed (31%) could undergo salvage by shunt surgery. Although hepatic portal perfusion was better maintained after sclerotherapy, there were no major differences between the groups in terms of post-therapy hepatic or psychoneurologic function.**

**In a predominantly alcoholic cirrhotic patient population (half non-urban), the results of elective shunt surgery were superior to those of chronic endoscopic sclerotherapy with respect to the prevention of recurrent variceal hemorrhage and survival.**

KEY WORDS: Distal splenorenal shunt, sclerotherapy, portal hypertension, oesophageal varices, variceal bleeding, cirrhosis

### PAPER DISCUSSION

In the 1990's, endoscopic sclerosis of esophageal varices and decompressive shunts are two of the many therapies required to provide optimum management of patients with variceal bleeding. This paper from Rikkers and his colleagues is an important contribution in helping to define the places of these therapies. This prospective randomized controlled trial reports the status of all patients entered, has a mean 7 year follow up, and presents objective data. As such, it should receive a heavy positive weighting in consideraation of studies for management of variceal bleeding. What are the major lessons it teaches us?

The patient population is an important factor in outcome and therapy choice. Two features of the patient population in this study influence the outcome. Eighty seven percent of patients randomized had alcoholic cirrhosis, and 57% of those who survived more than 3 months returned to drinking. Outcome is applicable to this group of patients, and should not be extrapolated to patients with other etiologies of their portal hypertension. In addition, this study was conducted in two geographic locations with a wide dispersion of patients in each location. In the group randomized to sclerotherapy geographic factors probably played a role in outcome in that 27% died of rebleeding, with delayed access to "surgical rescue" playing a role in that mortality. The lesson: sclerotherapy is not a good choice for those who live in remote locations with poor access to medical care.

Long-term management of variceal bleeding with sclerotherapy is associated with a high risk of rebleeding. In this study, with 7 year follow up, 60% of patients rebleed through sclerotherapy with 43% of the total group failing sclerotherapy. This failure was defined as death from rebleeding or the need for surgery for bleeding. This observation is consistent with other randomized studies in which sclerotherapy has been a treatment option and in which there is long-term follow up (1, 2, 3). While the greatest risk of rebleeding through sclerotherapy is early in the course—the risk never goes away. In this study the authors do further emphasize that the later rebleeding is frequently from gastric varices or portal hypertensive gastropathy. In contrast, decompressive shunt provides significantly better control of bleeding, which in this study translated to an improved survival. The lessons: i) rebleeding through sclerotherapy is about 50% at 5 years, and ii) prevention of rebleeding is one variable in improving survival.

Liver function and encephalopathy were carefully followed in this study. Quantita-tive liver function measured by galactose elimination capacity and indocyanine green clearance, and hepatic encephalopathy measured by clinical, psychometric and electroencephalographic studies were not significantly different in the two randomized groups. While at first sight one is tempted to say that the disadvantages of rebleeding through sclerotherapy and loss of portal perfusion in some of the shunt patients offset each other, this does not really appear to be the case. No patients had severe disabling encephalopathy, and the quantitative measures of liver function remained good. These data are consistent with our own study comparing DSRS and sclerotherapy[1] which measured quantitative liver function. We showed that patients successfully managed with sclerotherapy, who had no major rebleeding, showed a significant improvement in galactose elimination capacity over the first year[1]. Lesson: the data in this study show that neither of the randomized therapies significantly accelerate liver failure compared to each other.

What of the high mortality and failure to achieve surgical rescue of patients who failed sclerotherapy in this study? This factor is the major difference between this study and our previously published study[1]. In the Emory study only one patient randomized to sclerotherapy died as a direct result of rebleeding, and 12 were successfully salvaged by surgical management. In contrast, in the present study 8 patients died as a direct result of rebleeding in the sclerotherapy group and only 5 patients had surgical rescue. The authors correctly point out that access was a major problem for their sclerotherapy patients who rebleed, and this highlights one of the shortcomings of sclerotherapy. What are the lessons? In patients in whom sclerotherapy is selected as primary management, a strategy should be in place from the outset to treat rebleeding or consider surgical treatment for persistent high risk varices.

Finally, it must be remembered that sclerotherapy and surgical shunt are not the only treatments for variceal bleeding. Liver transplant has dramatically altered the management of patients with end-stage liver disease. But, it is end-stage liver disease and not variceal bleeding per se which is the indication for transplant. However, in any patient who bleeds from varices, full evaluation at the time of the initial bleed is a critical step to help answer the question: is this patient now, or will they in the future be a candidate for liver transplant? The answer to that question will influence treatment choice. We have recently published an approach to the evaluation of such patients[4]. In addition, the radiologists are back in the fray[5]. Transjugular intrahepatic portal system shunts (TIPS) can provide portal decompression, but lack of randomized trials and good objective data leave the role of TIPS to be defined.

In summary, the four prospective randomized trials which have compared DSRS to sclerotherapy all show better control of bleeding with decompressive shunt[6]. Neither therapy appears to significantly accelerate liver failure. Survival differs in these studies, and the difference depends entirely on how patients who fail sclerotherapy are managed. The important factors in this are the type of patient, their access to care, and the type of care available to the patient. To the physician making management decisions, forward planning with these factors in mind is the key.

*References*

1. Henderson, J. M., Kutner, M. H. and Millikan, W. J. *et al.* (1990) Endoscopic variceal sclerosis compared with distal splenorenal shunt to prevent recurrent variceal bleeding in cirrhosis. A prospective randomized trial. *Ann. Int. Med.*, **112**, 262–269.
2. Terblanche, J., Bornmann, P. C. and Kahn, D. *et al.* (1983) Failure of repeated injection sclerotherapy to improve long-term survival after oesophageal variceal bleeding. A five year prospective controlled clinical trial. *Lancet* **2**, 1328–1332.
3. Westaby, D. and Williams, R. (1990) Status of sclerotherapy for variceal bleeding in 1990. *Am. J. Surg.*, **160**, 32–37.
4. Henderson, J. M., Gilmore, G. T. and Hooks, M. A. *et al.* (1992) Selective shunt in the management of variceal bleeding in the era of liver transplant. *Ann. Surg.*, **216**, 248–255.
5. Conn, H. O. (1993) Transjugular intrahepatic portal-systemic shunts: The state of the art. *Hepatology* **17**, 148–158.
6. Spina, G. P., Henderson, J. M. and Rikkers, L.F. *et al.* (1992) Distal splenorenal shunt versus endoscopic sclerotherapy in the prevention of variceal rebleeding. A meta-analysis of 4 randomized clinical trials. *J. Hepatology* **16**, 338–345.

J. Michael Henderson
Department of General Surgery
The Cleveland Clinic Foundation
9500 Euclid Avenue
Cleveland, Ohio 44195

### ENUCLEATION FOR GIANT LIVER HAEMANGIOMA

### ABSTRACT

*Baer, H. U., Dennison, A. R., Mouton, W., Stain, S. C., Zimmerman, A. and Blumgart, L H. (1992) Enucleation of giant hemangiomas of the liver. Annals of Surgery, 216, 673–676.*

